# Hormonal regulation of the hypothalamic melanocortin system

**DOI:** 10.3389/fphys.2014.00480

**Published:** 2014-12-09

**Authors:** Jung D. Kim, Stephanie Leyva, Sabrina Diano

**Affiliations:** ^1^Departments of Obstetrics, Gynecology and Reproductive Sciences, Yale University School of MedicineNew Haven, CT, USA; ^2^Program in Cell Signaling and Neurobiology of Metabolism, Yale University School of MedicineNew Haven, CT, USA; ^3^Department of Neurobiology, Yale University School of MedicineNew Haven, CT, USA; ^4^Section of Comparative Medicine, Yale University School of MedicineNew Haven, CT, USA

**Keywords:** hypothalamic melanocortin system, arcuate nucleus, pro-opiomelanocortin (POMC), neuropeptide Y (NPY), agouti-related peptide (AgRP), hormones, obesity

## Abstract

Regulation of energy homeostasis is fundamental for life. In animal species and humans, the Central Nervous System (CNS) plays a critical role in such regulation by integrating peripheral signals and modulating behavior and the activity of peripheral organs. A precise interplay between CNS and peripheral signals is necessary for the regulation of food intake and energy expenditure in the maintenance of energy balance. Within the CNS, the hypothalamus is a critical center for monitoring, processing and responding to peripheral signals, including hormones such as ghrelin, leptin, and insulin. Once in the brain, peripheral signals regulate neuronal systems involved in the modulation of energy homeostasis. The main hypothalamic neuronal circuit in the regulation of energy metabolism is the melanocortin system. This review will give a summary of the most recent discoveries on the hormonal regulation of the hypothalamic melanocortin system in the control of energy homeostasis.

## Hypothalamic melanocortin system

Energy homeostasis is a tightly regulated process and the imbalance between its components, food intake and energy expenditure, causes metabolic dysfunctions including obesity, a major risk factor for comorbidities including type 2 diabetes, hypertension and stroke, and cardiovascular diseases. A major player in the regulation of energy homeostasis is the Central Nervous System (CNS) and specifically the hypothalamus. By monitoring, processing and responding to peripheral signals, such as hormones, the hypothalamus in turn will regulate peripheral organ functions. Within the hypothalamus several neuronal populations have been identified as important players in metabolism regulation. The central melanocortin system consists of three neuronal populations: the pro-opiomelanocortin (POMC)-expressing neurons, the neuropeptide Y (NPY) and agouti-related peptide (AgRP)-co-expressing neurons (Cowley et al., 1999; Elmquist et al., 1999) located in the hypothalamic arcuate nucleus and the melanocortin 4 receptor (MC4R)-expressing neurons located in the hypothalamic paraventricular nucleus. While the anorexigenic POMC neurons, by activating MC4R neurons, induce decreased food intake and increased energy expenditure, the orexigenic NPY/AgRP neurons, by antagonizing POMC action on MC4R, increase food intake and decrease energy expenditure, thus increasing body weight.

The POMC gene encodes a protein precursor that generates a number of bioactive peptides, including adrenocorticotrophin (ACTH), α-, β-, and γ-melanocyte stimulating hormone (α-MSH, β-MSH, and γ-MSH) and β-endorphin, via several post-translational modification processes. Among these peptides, α-MSH is the most well-known anorexigenic peptide which mediates the effects on food intake and energy expenditure through its binding and activation of MCRs (Ollmann et al., 1997).

The critical role of POMC in the regulation of metabolism has been evidenced by studies showing that in humans, individuals with POMC gene mutations display early-onset obesity (Krude et al., 1998; Krude and Gruters, 2000). A similar obese phenotype occurs also in POMC-deficient mice (Yaswen et al., 1999). However, while the acute ablation of POMC neurons in adult mice results in an obese phenotype with hyperphagia (Gropp et al., 2005), postnatal ablation of POMC neurons leads to an obese phenotype with reduced energy expenditure but no hyperphagia (Greenman et al., 2013). On the other hand, reactivation of central POMC at different stages of development in neural-specific POMC deficient mice reduces food intake and weight gain and attenuates comorbidities such as hyperglycemia and hyperinsulinemia (Bumaschny et al., 2012).

Contrary to POMC mutations, NPY, and AgRP gene mutations produce a negligible phenotype with no difference in food intake and body weight (Erickson et al., 1996; Qian et al., 2002; Gropp et al., 2005; Luquet et al., 2005), suggesting that compensatory mechanisms may occur during development (Wu and Palmiter, 2011). Indeed, with the use of the diphtheria toxin and diphtheria toxin receptor (DTR)-mediated cell specific knockout system (Saito et al., 2001), acute ablation of AgRP neurons in adult mice causes a significant metabolic effect (Gropp et al., 2005; Luquet et al., 2005). Because these neurons also produce and release the inhibitory amino acid neurotransmitter, γ-aminobutyric acid (GABA), its role in the development of this metabolic phenotype regulation has been postulated. Indeed, AgRP-specific vesicular GABA transporter knockout mice have a lean phenotype and are resistant to high fat diet-induced obesity (Tong et al., 2008). GABAergic signaling by NPY/AgRP neurons inhibits POMC neurons in the ARC by direct synaptic innervations (Horvath et al., 1997; Cowley et al., 2001) and also inhibits the parabrachial nucleus (PBN) in the hindbrain (Wu et al., 2009). Moreover, the direct delivery of bretazenil (a GABA receptor partial agonist) into the PBN prevented anorexia caused by AgRP neuronal ablation, suggesting that GABAergic signaling in the PBN is important for the regulation of feeding (Wu et al., 2009).

Recently, advanced technologies such as the optogenetic and DREADD (designer receptors exclusively activated by designer drugs) systems have provided stronger evidences on the central role of POMC and NPY/AgRP neurons in the regulation of feeding behavior (Aponte et al., 2011; Krashes et al., 2011, 2013; Atasoy et al., 2012; Zhan et al., 2013). For example, Zhan and collaborators recently showed that POMC neurons regulate feeding by integrating long-term information from the ARC and short-term information from the NTS in the brainstem (Zhan et al., 2013). On the other hand, activation of AgRP neurons acutely promotes feeding behavior but suppression of POMC neurons is not required for this acute effect (Aponte et al., 2011; Krashes et al., 2011; Atasoy et al., 2012). One study, using DREADD system, has addressed the temporal effect of the acute activation of AgRP neurons on food intake in single, double or triple knockdown of NPY, AgRP-specific vesicular GABA transporter and MC4R (Krashes et al., 2013). This study proposes that NPY and GABA are required for the short-term feeding response while AgRP is responsible for the long-term feeding response.

α-MSH and AgRP exert their metabolic effects through direct interaction with MCRs. In the CNS, MC3R, expressed in the hypothalamic arcuate POMC nucleus, and MC4R, expressed in several brain areas including the paraventricular nucleus of the hypothalamus, play a critical role in mediating the agonistic and antagonistic effect of α-MSH and AgRP, respectively (Ollmann et al., 1997). Both MC3R and MC4R deficient mice displayed an obese phenotype, however, while MC3R are not hyperphagic (Butler et al., 2000; Chen et al., 2000; Renquist et al., 2012), MC4R deficiency mice show hyperphagia and reduced energy expenditure (Huszar et al., 1997; Marsh et al., 1999; Butler et al., 2001). In addition, mutations in the human *MC4R* gene are associated with non-syndromic obesity (Vaisse et al., 1998; Yeo et al., 1998).

The importance of MC4R-expressing neurons in the PVN in regulating metabolism has been recently showed by studies in which restoration of MC4Rs in PVN Single-minded 1 (SIM1) neurons of MC4Rs deficient mice induced a reduced obese phenotype (Xu et al., 2013; Shah et al., 2014). Of note, SIM1 seems to play also an important role in metabolism regulation both in humans and mice (Holder et al., 2000; Michaud et al., 2001).

The activity of the melanocortin neurons is regulated by many peripheral signals including hormones such as leptin, ghrelin, insulin, glucocorticoids, and thyroid hormones. By either activating or inhibiting these neurons, these peripheral signals convey information on the metabolic status of the organism.

## Leptin

Leptin is an anorexigenic hormone produced and released by the white adipose tissue in the amount proportional to the mass of fat in the body (Zhang et al., 1994; Frederich et al., 1995; Maffei et al., 1995). Leptin interacts with six types of receptors (*Ob-Ra, -Rb, -Rc, Rd, Re, and Rf*) encoded by a single leptin receptor gene (*Ob-R*) (Lee et al., 1996; Wang et al., 1996). However, only the long form of leptin receptors (*Ob-Rb*), consisting of an extracellular and an intact cytoplasmic domain, mediates the anorexigenic effect of leptin (De Luca et al., 2005). Deficiency in leptin or leptin receptors induces a morbid obese phenotype characterized by hyperphagia, hyperglycemia, hyperlipidemia, and reduced energy expenditure in both rodents and humans (Halaas et al., 1995; Chen et al., 1996; Montague et al., 1997; Clement et al., 1998). Neuron-specific Ob-R-deficient (*db/db*) mice displayed an obese phenotype while hepatocyte-specific *db/db* mice are normal (Cohen et al., 2001; De Luca et al., 2005), suggesting that the direct effect of leptin in the brain is essential for metabolism regulation. Mice lacking Ob-Rb in POMC neurons, AgRP neurons or both POMC/AgRP neurons displayed increased body weight and fat mass (Balthasar et al., 2004; Van De Wall et al., 2008), although their phenotypes are milder compared to those of whole brain neuron-specific Ob-Rb deficient mice, suggesting the involvement of other leptin receptor-expressing neurons in mediating the effect of leptin on energy homeostasis.

Leptin regulates POMC and NPY/AgRP neurons at different levels. For examples, leptin increases POMC mRNA levels while decreasing NPY/AgRP mRNAs (Mizuno et al., 1998; Mizuno and Mobbs, 1999). Besides the transcriptional regulation, leptin directly depolarizes (activates) POMC neurons while simultaneously hyperpolarizing (inactivates) NPY/AgRP neurons (Cowley et al., 2001). In addition, systemic leptin administration to leptin deficient (ob/ob) mice rapidly induced synaptic input reorganization onto NPY/AgRP and POMC neurons (Pinto et al., 2004), suggesting that the synaptic rearrangement is an important event for leptin-induced behavioral changes (Horvath, 2006). A recent study demonstrated that AgRP neurons are critical in mediating metabolic syndrome in ob/ob mice since ablation of Agrp neurons in leptin deficient (*ob/ob*) mice showed reduced food intake and improved glucose tolerance (Wu et al., 2012).

## Insulin

Insulin, produced from pancreatic β-cells, plays a fundamental role in the regulation of glucose homeostasis by modulating glucose uptake in peripheral organs (Bagdade et al., 1967). However, since insulin receptors (IR) are widely expressed in the brain (Havrankova et al., 1978), accumulated evidences have indicated a role for insulin in the CNS. Indeed, brain specific-IR deficient mice develop obesity with increased body weight, fat mass, and food intake (Bruning et al., 2000). However, mice with selective deletion of IR in the arcuate melanocortin system, either POMC or AgRP neurons, did not display alteration in energy homeostasis, while only a defective suppression on hepatic glucose production was found in mice with selective deletion of IR in AgRP neurons (Konner et al., 2007). In addition, re-expression of IR in either POMC or AgRP neurons of L1 mice, a genetic mouse model with significant reduction of IR in the ARC (Okamoto et al., 2004), suggested that insulin signaling in AgRP neurons negatively regulates hepatic glucose production while IR activation in POMC neurons positively regulates hepatic glucose production and energy expenditure (Lin et al., 2010). Interestingly, when both leptin and IR were ablated in POMC neurons, systemic insulin resistance despite increased pancreatic insulin secretion was observed in these mice (Hill et al., 2010). In addition, the obese phenotype observed in POMC-specific ObR knockout mice was ameliorated when both ObR and IR were selectively deleted from POMC neurons, suggesting that insulin and leptin signaling in POMC neurons may have opposing effects in the regulation of body weight but additive effects on glucose homeostasis. In support of this, leptin and insulin responsive neurons are expressed in distinct subpopulations of POMC neurons (Williams et al., 2010). Thus, the divergent effects of leptin and insulin on energy homeostasis may due to the activation of different POMC subpopulations.

## Ghrelin

Ghrelin, the hunger hormone, is predominantly secreted by specialized endocrine cells of the stomach when the stomach is empty. Ghrelin is synthesized as a preprohormone and several processes are required to generate its active form. After removing the signal peptide from the preprohormone, ghrelin precursor is acylated at the third serine with n-octanoic acid by an enzyme called ghrelin o-acyltransferase (GOAT) (Gutierrez et al., 2008; Yang et al., 2008) and then it is cleaved by prohormone convertase 1/3 to produce the active 28-amino-acid acylated ghrelin (Zhu et al., 2006). Ghrelin exerts its orexigenic effects through the growth hormone secretagogue receptor (GHSR) (Sun et al., 2004). The physiological function of Ghrelin and GHSR on feeding has been demonstrated by ghrelin and GHSR deficient mice. Both ghrelin- and GHSR-deficient mice are resistant to high-fat diet-induced obesity but only when mice are exposed to the diet shortly after post-weaning and not in adulthood (Sun et al., 2003; Wortley et al., 2004, 2005; Zigman et al., 2005).

The strongest expression of GHSR has been observed in the hypothalamus (Willesen et al., 1999). This observation, together with ghrelin-induced high c-fos expression in the hypothalamus, indicated this site of the CNS as main site of ghrelin's action. In the ARC, GHSR is predominantly expressed in NPY/AgRP neurons. Accordingly, peripheral and central administration of ghrelin induced c-fos expression in NPY/AgRP neurons (Nakazato et al., 2001; Wang et al., 2002). In addition, ghrelin increases NPY and AgRP mRNA expression levels and the electrical activity of NPY/AgRP neurons (Kamegai et al., 2001; Shintani et al., 2001; Cowley et al., 2003; Seoane et al., 2003; Van Den Top et al., 2004). Although ghrelin inhibits POMC neuronal activity, no GHSR expression has been reported in POMC neurons (Willesen et al., 1999) thus suggesting that this inhibitory effect may be mediated by the activation of NPY/AgRP neurons. In addition, ghrelin positively regulates prolyl carboxypeptidase, the enzyme responsible for α-MSH degradation (Kwon Jeong et al., 2013) thus further increasing the orexigenic tone. Ghrelin-induced food intake is mediated by NPY/AgRP neurons (Nakazato et al., 2001; Shintani et al., 2001; Chen et al., 2004). For example, administration of neutralizing antibodies or antagonists of both NPY and AgRP blunted the orexigenic effects of ghrelin. Consistently, ghrelin's effect on feeding is abolished in NPY/AgRP double-deficient mice (Chen et al., 2004). NPY/AgRP neurons-mediated ghrelin's effect is evident in diet-induced obese mice (DIO). DIO mice show decreased expression of *Npy* and *AgRP* mRNA levels, decreased *Goat* mRNA levels in the stomach and decreased hypothalamic *GHSR* expression and they are ghrelin resistance (Briggs et al., 2010). Interestingly, while ghrelin resistance has been observed in DIO mice, *ob/ob* mice retain their sensitivity to ghrelin, suggesting that elevated leptin levels may be involved in the development of ghrelin resistance. In support of this, a recent study showed that central leptin administration in *ob/ob* mice induced ghrelin resistance (Briggs et al., 2014). AgRP-selective re-expression of GHSR in whole body of GHSR-deficient mice partially restores the orexigenic response to ghrelin (Wang et al., 2014). In addition, AgRP-specific vesicular GABA transporter knockout mice showed impaired ghrelin-mediated feeding response and impaired inhibitory postsynaptic potentials in POMC neurons, indicating that GABA release from AgRP neurons is an important mediator of ghrelin's effect on food intake (Tong et al., 2008). Of note, uncoupling protein 2 (UCP2) is a mediator of ghrelin's action on feeding behavior in both the hypothalamus and the ventrotegmental area (VTA). In *Ucp2* deficient mice, intrahypothalamic administration of ghrelin showed a blunted effect on food intake and intra-VTA ghrelin injection also attenuated food intake (Andrews et al., 2008).

## Thyroid hormones

Thyroid hormones play an important role in metabolism regulation affecting nearly all the tissues in the body. Thyroxine (T4) produced by the thyroid gland is converted in target tissues in the active form of thyroid hormone, Triiodothyronine (T3), by a process called 5′ deiodination. Thyroid hormones affect metabolism acting on both food intake and energy expenditure (Vijayan and Mccann, 1977; Suzuki et al., 1982; Lin et al., 1983; Choi et al., 2002; Herwig et al., 2008; Klieverik et al., 2009). The hyperphagia, induced by increased thyroid hormones levels, is mediated by the CNS. Indeed, central T3 administration induced increased food intake by increasing and reducing NPY and POMC mRNA levels, respectively (Ishii et al., 2003). Changes in central T3 levels occur during different metabolic states (Van Haasteren et al., 1995). For example, elevated levels of T3 occur in the hypothalamus during starvation (Coppola et al., 2005, 2007). This increased T3 is due to the elevated activity of the enzyme responsible for the conversion of T4 in T3 (Diano et al., 1998). Similar to the effect of T3 in the brown adipose tissue in increasing uncoupling protein 1 (UCP1) activity, T3 in the hypothalamus regulates UCP2 levels, which in turn, will affect the activity of NPY/AgRP neurons and thus increase food intake. Via this signaling pathway involving NPY/AgRP neuronal activity, T3 is also responsible for fasting-induced suppression of TRH mRNA expression in the PVN (Coppola et al., 2005, 2007; Vella et al., 2011).

## Glucocorticoids

Glucocorticoids are known regulators of energy balance (Nieuwenhuizen and Rutters, 2008). For example, Cushing' syndrome, one pathology characterized by hypercortisolism, displays several symptoms including hypertension, insulin resistance, hyperglycemia as well as rapid weight gain (Hankin et al., 1977). Conversely, Addison's disease, condition of hypocortisolism, causes weight loss (Lovas and Husebye, 2007). Similarly, hypocortisolism induced by adrenalectomy (ADX) reduces food intake, fat stores and body weight (Dallman et al., 2004). Since ACTH, which is generated from POMC-expressing cells in the pituitary gland, stimulates the production of glucocorticoids from the adrenals, pituitary POMC's effects on energy balance were addressed in neural-specific POMC deficient mice (Smart et al., 2006). Interestingly, increased glucocorticoids by re-expression of pituitary POMC in neural-specific POMC deficient mice exacerbates obesity with severe insulin resistance, suggesting that central POMC's role is not substituted by peripheral POMC (Smart et al., 2006). In further support of the role of glucocorticoids in the regulation of energy balance, many obese mouse models including diet-induced obesity and genetic mouse models were characterized by elevated corticosterone levels, and ADX to these mice has been shown to ameliorate their obese phenotype (Okada et al., 1993; Makimura et al., 2000). The mechanism by which ADX ameliorates obesity involves the CNS. For example, corticosterone has been shown to affect the hypothalamic melanocortin signaling by regulating POMC and AgRP mRNA expression in leptin-deficient mice (Makimura et al., 2000). In addition, studies from our group have shown that ADX directly influences neuronal activity of the arcuate POMC neurons by affecting the synaptic input organization of these neurons (Gyengesi et al., 2010). Furthermore, ADX has been reported to enhance leptin's effect on feeding by reducing the responsiveness of melanocortin receptors to its ligands (Drazen et al., 2003).

## Conclusions

The hypothalamic melanocortin system is an integrative center in the regulation of energy balance. Numerous studies have shown that many peripheral signals including hormones can directly influence the activity of the hypothalamic melanocortin system. Besides hormones, other signals including nutrients such as glucose (Thorens, 2012), lipids (Lam et al., 2005; Moulle et al., 2014), and amino acids (Cota et al., 2006; Schwartz, 2013) also function as signal molecules for the melanocortin system.

All of these signals have been shown to regulate the melanocortin system via extracellular and intracellular morphological changes that will affect the activity levels of the different component of the system. These changes include synaptic input organization (Pinto et al., 2004; Zeltser et al., 2012), neuron-glia interaction, and intracellular organelles alterations including mitochondrial and peroxisomal density and function (for review see Koch and Horvath, 2014).

In conclusion, further studies on the mechanisms controlling the melanocortin system are crucial to make advancement in our abilities to develop new therapies for the treatment of metabolic disorders.

### Conflict of interest statement

The authors declare that the research was conducted in the absence of any commercial or financial relationships that could be construed as a potential conflict of interest.
